# The pediatric respiratory assessment measure as a predictor of hospitalization in the management of asthma exacerbations: an observational study^☆^

**DOI:** 10.1016/j.jped.2026.101583

**Published:** 2026-07-24

**Authors:** Leopoldo Silocchi Pergo, Paula Adamo de Almeida, Gabriel Bordignon, Luis Eduardo Martins Bertoli, Solena Ziemer Kusma Fidalski, Débora Carla Chong-Silva, Rebeca Amelia Toassa Gomes Mousquer

**Affiliations:** a Department of Pediatrics, Hospital Pequeno Príncipe, Curitiba, PR, Brazil; b Postgraduate Program in Public Health, Universidade Federal do Paraná, Curitiba, PR, Brazil; c Pediatric Pulmonology Department, Hospital Pequeno Príncipe, Curitiba, PR, Brazil

**Keywords:** Asthma, Pediatrics, Bronchial spasm, Emergency medicine, Patient outcome assessment, Risk assessment

## Abstract

**Objective:**

To evaluate the Pediatric Respiratory Assessment Measure (PRAM) score for hospital admission and prolonged stay risk stratification in the emergency department (ED) at different time points.

**Method:**

Retrospective cohort study using prospectively collected protocol data (August 2023–March 2025) at a tertiary pediatric ED. Patients aged 2–17 years with acute asthma exacerbations were included; those with systemic comorbidities, other chronic lung diseases, or confounding acute conditions were excluded. PRAM was assessed hourly (PRAM 0 to 4). Outcomes were hospital admission and prolonged ED stay (> 3 h).

**Results:**

A total of 428 visits were analyzed (35.0% hospital admission rate). PRAM demonstrated satisfactory discriminatory capacity for admission at triage (AUC: 0.791; 95% CI: 0.745–0.836; p < 0.001), with optimized accuracy during serial assessments, performing best at the second hour (PRAM 2; AUC: 0.829; 95% CI: 0.773–0.885; p < 0.001). For predicting prolonged ED stay, the score showed moderate accuracy at the second hour (AUC: 0.677; p < 0.001), with no significant correlation between sequential scores and time to admission decision (p > 0.05).

**Conclusions:**

PRAM is a valuable, dynamic risk-stratification tool for pediatric asthma exacerbations. Serial assessments provide superior prognostic accuracy compared to a single static measurement at admission by actively reflecting therapeutic response. Although its ability to predict operational outcomes is restricted by non-clinical factors, the score demonstrates consistent utility for standardizing clinical assessment and supporting medical disposition in the ED.

## Introduction

Asthma is a heterogeneous chronic inflammatory disease of the lower airways characterized by bronchial hyperresponsiveness and variable airflow limitation [1,2], affecting over 300 million people worldwide as the most prevalent childhood chronic condition [2]. In Brazil, its impact on the public healthcare system is substantial, historically ranking as a leading cause of pediatric hospitalization [3]. Recent DATASUS data reveal a concerning upward trend in mortality, peaking at 2802 deaths in 2022 and 2611 in 2023 [3] while hospital costs in the public system exceeded BRL 265 million in 2023 [3]. Acute exacerbations drive most of these expenditures [4], rapidly translating into severe clinical signs in children due to their small airway caliber and low ventilatory reserve [2,5]. This scenario became more complex between 2022 and 2025 due to a post-pandemic surge in viral circulation and more severe presentations related to the pediatric *'immunity debt'* [3,[5], [6], [7]].

Given the clinical variability and increased severity of asthma exacerbations, subjective assessment is associated with significant inter-observer inconsistency. Therefore, objective tools are recommended by international guidelines, such as the Global Initiative for Asthma (GINA, 2026) [2], to provide a reliable substrate for clinical decision-making. The Pediatric Respiratory Assessment Measure (PRAM) stands out as a standardized tool validated for children aged 2 to 17 years [[8], [9], [10], [11]]. PRAM evaluates five physiological parameters—suprasternal retractions, scalene muscle contraction, air entry, wheezing, and oxygen saturation—assigning total scores from 0 to 12 [[8], [9], [10]], which classify exacerbations as mild (0–4), moderate (5–8), or severe (9–12) [[8], [9], [10],^12^]. Recent evidence reinforces PRAM's utility not only as a cross-sectional baseline tool but also as a dynamic predictor, where sequential reassessments at the 3rd or 4th hour of care carry high predictive value for hospital admission [6,11].

Therefore, this study aims to evaluate the performance of the PRAM score as a risk stratification tool for hospital admission and prolonged stay in the emergency department in children treated for acute asthma exacerbations at a tertiary pediatric hospital, analyzing its accuracy across multiple time points.

## Methods

This retrospective cohort study, based on a prospectively implemented clinical protocol, was conducted between August 2023 and March 2025 at the public Emergency Department of a tertiary teaching pediatric hospital [blinded for review]. Eligible visits were retrospectively identified from electronic medical records using the ICD-10 code J45.9 (unspecified asthma). Although data extraction was retrospective, all clinical parameters and PRAM scores were recorded in real-time by the medical team during patient care. The center acts as a regional tertiary reference for high-complexity pediatric emergencies, managing both spontaneous admissions and regulated transfers from pre-hospital networks.

Patients aged 2 to 17 years with a prior or new diagnosis of asthma made during the emergency visit were included. Exclusion criteria comprised hypersensitivity to oral corticosteroids, other chronic lung diseases (e.g., bronchopulmonary dysplasia, cystic fibrosis), significant systemic comorbidities (metabolic, cardiac, neurological, or immunological), a history of adrenal suppression, recent varicella exposure, or coexisting acute respiratory conditions (e.g., laryngitis, pneumonia, anaphylaxis). The primary variables analyzed were the sequential PRAM scores, clinical disposition (hospital admission or emergency department discharge), and a prolonged emergency stay, operationally defined as an interval exceeding three hours until the final medical decision. For the purposes of this study, *'clinical decision'* was operationally defined as the exact moment the attending physician recorded the final disposition — either hospital admission or discharge instructions — in the patient's electronic medical record (EMR). This definition was chosen to minimize potential bias related to boarding times or administrative delays in physical transfer from the emergency department.

As an integral part of the institutional protocol, the PRAM score was recorded in real-time by unblinded residents and staff physicians for clinical monitoring. To mitigate bias, the score served as a clinical aid rather than a deterministic tool, leaving final disposition to the attending physician's judgment. PRAM was applied at first contact (PRAM 0) and reassessed hourly up to the fourth hour of care (PRAM 1 to PRAM 4). For categorical analysis, scores were bifurcated into a binary variable: mild (≤ 4 points) versus moderate-to-severe (≥ 5 points).

Baseline vital signs — including peripheral oxygen saturation (SpO_2_) and axillary temperature — were recorded at triage immediately upon arrival, prior to any intervention. The institutional protocol for asthma exacerbations established first-hour management consisting of up to three cycles of inhaled salbutamol (every 20 min) combined with systemic corticosteroid administration (oral prednisolone 1 mg/kg/dose). Subsequent reassessments, potential dose repetitions, and secondary therapies—such as intravenous medications or supplemental oxygen—were guided by clinical evolution and sequential PRAM scores. Following this initial management protocol, a total emergency department stay exceeding three hours until the final medical disposition was defined as a prolonged stay.

Data were retrospectively extracted from electronic medical records using the ICD-10 code J45.9. The study relied on a convenience sample of all eligible visits during a 19-month period, without an a priori sample size calculation. To account for the intra-patient dependency of multiple visits (clustering effect), generalized linear mixed models (GLMM) with a binomial distribution, logit link function, and a random intercept for patient ID were utilized to analyze the binary outcomes of hospital admission and prolonged stay (> 3 h).

Continuous variables were expressed as mean ±SD or median (IQR), and categorical variables as frequencies. Separate univariable and multivariable GLMMs were fitted for initial PRAM intervals (PRAM 0, 1, and 2) using mild scores (≤ 4 points) as the reference category; adjustment variables were selected based on univariable significance and clinical relevance to minimize collinearity. Discriminatory performance was evaluated using Receiver Operating Characteristic (ROC) curves to calculate areas under the curve (AUC) and 95% confidence intervals. Spearman’s coefficient assessed the correlation between sequential PRAM scores and time to admission. Missing data from progressive dropouts were related to clinical endpoints (informative censoring) and thus analyzed without imputation. Analyses were performed using SPSS version 32.0, with significance set at 5% (p < 0.05).

The study was submitted to and approved by the Research Ethics Committee [*blinded for review]* under CAAE protocol number 69784423.0.0000.0097.

## Results

A total of 428 emergency department (ED) visits from 359 unique pediatric patients with asthma were included. Most patients contributed a single visit (n = 302, 84.1%), while 57 patients (15.9%) contributed multiple entries. Sequential PRAM assessments were available for all 428 visits at admission (PRAM 0), dropping to 339 visits at 1 hour (20.8% missing data), 210 visits at 2 h (50.9% missing data), 113 visits at 3 h (73.6% missing data), and 11 visits at 4 h (97.4% missing data), reflecting patient discharge or admission over time.

The baseline demographic and clinical characteristics of the sample, stratified by hospital admission and ED length of stay, are summarized in Table 1. Briefly, the sample had a median age of 6 years (IQR: 4–8) and a slight male predominance (55.4%). At admission, an associated upper respiratory tract infection was present in 48.6% of the visits, a personal history of atopy in 54.7%, and a history of previous hospital and ICU admissions for asthma in 40.7% and 29.3%, respectively. Upon triage, the baseline median SpO2 was 93% (IQR: 90–96), and 48.0% of the patients required supplemental oxygen during their stay. Acute ED management strategies and previous medication use are also detailed in Table 1.

**Table 1:** Clinical and demographic profile of visits according to hospital admission and length of stay.

**Variables**	**Total (n=428)**	**Hospital admission**			**Stay**		
		**No**	**Yes**	p*	**≤ 3h**	**> 3h**	p*
Age (years) (mean ± sd)	6.3 ± 3.2	6.3 ± 3.2	6.3 ± 3.1	0.898	6.2 ± 3.1	6.4 ± 3.2	0.544
Male sex, n (%)	237 (55.4%)	163 (58.6%)	74 (49.3%)	0.076	91 (53.8%)	146 (56.4%)	0.595
Duration of previous symptoms (days) (median; IQR)	1 (1 - 3)	2 (1 - 3)	1 (1 - 2)	0.038	2 (1 - 3)	1 (1 - 3)	0.331
Associated upper respiratory tract infection, n (%)	208 (48.6%)	134 (48.2%)	74 (49.3%)	0.858	85 (50.3%)	123 (47.5%)	0.564
Personal history of atopy, n (%)	234 (54.7%)	157 (56.5%)	77 (51.3%)	0.315	87 (51.5%)	147 (56.8%)	0.297
Family history of atopy, n (%)	162 (37.9%)	109 (39.4%)	53 (35.3%)	0.430	72 (42.6%)	90 (34.9%)	0.119
Previous use of salbutamol, n (%)	258 (60.3%)	171 (61.5%)	87 (58.0%)	0.467	93 (55.0%)	165 (63.7%)	0.082
Previous use of inhaled corticosteroids (> 2 weeks), n (%)	177 (41.4%)	121 (43.5%)	56 (37.3%)	0.221	77 (45.6%)	100 (38.6%)	0.159
Use of inhaled corticosteroids in current crisis, n (%)	131 (30.6%)	76 (27.3%)	55 (36.7%)	0.049	73 (43.2%)	58 (22.4%)	<0.001
Previous use of montelukast, n (%)	132 (30.8%)	80 (28.8%)	52 (34.7%)	0.217	69 (40.8%)	63 (24.3%)	<0.001
Previous use of antibiotics, n (%)	145 (33.9%)	91 (32.7%)	54 (36.0%)	0.481	77 (45.6%)	68 (26.3%)	<0.001
Previous hospital admission for asthma, n (%)	174 (40.7%)	104 (37.5%)	70 (46.7%)	0.079	76 (45.0%)	98 (38.0%)	0.158
Previous ICU admission for asthma, n (%)	125 (29.3%)	74 (26.7%)	51 (34.0%)	0.123	71 (42.0%)	54 (20.9%)	<0.001
SpO2	92.6 ± 4.0	93.9 ± 3.3	90.2 ± 4.0	<0.001	93.5 ± 4.3	92.0 ± 3.6	<0.001
Salbutamol	283 (66.1%)	179 (64.4%)	104 (69.3%)	0.345	85 (50.3%)	198 (76.4%)	<0.001
Ipratroprium	151 (35.3%)	93 (33.5%)	58 (38.7%)	0.310	60 (35.5%)	91 (35.1%)	0.987
Oral Corticosteroids	238 (55.6%)	148 (53.2%)	90 (60%)	0.205	70 (41.4%)	168 (64.9%)	<0.001
Endovenous Corticosteroids	152 (35.5%)	76 (27.3%)	76 (50.7%)	<0.001	74 (43.8%)	78 (30.1%)	0.005
MgSO4	136 (31.8%)	72 (25.9%)	64 (42.7%)	<0.001	70 (41.4%)	66 (25.5%)	<0.001

ICU, intensive care unit; sd, standard deviation; IQR, interquartile interval.

Data expressed as mean ± standard deviation, median (interquartile interval), or frequency and percentages calculated based on the total number of valid cases for each variable;.

⁎ Generalized Linear Mixed Model (GLMM).

When analyzing the baseline and clinical characteristics according to the study outcomes, significant differences were observed. Regarding hospital admission, patients who required hospitalization had a shorter median duration of previous symptoms [1 day (IQR: 1–2) vs. 2 days (IQR: 1–3); p = 0.038] and a higher frequency of inhaled corticosteroid use during the current crisis (36.7%vs. 27.3%; p = 0.049). Hospitalized patients also presented significantly lower initial SpO2 values (90.2 ± 4.0% vs. 93.9 ± 3.3; p < 0.001) and required higher rates of intravenous corticosteroids (50.7%vs. 27.3%; p < 0.001) and magnesium sulfate (MgSO4) administration (42.7%vs. 25.9%; p < 0.001).

Regarding the length of stay in the emergency department, a prolonged stay (> 3 h) was significantly associated with a lower frequency of current inhaled corticosteroid use (22.4%vs. 43.2%; p < 0.001), previous montelukast use (24.3%vs. 40.8%; p < 0.001), previous antibiotic use (26.3%vs. 45.6%; p < 0.001), and previous ICU admission for asthma (20.9%vs. 42.0%; p < 0.001). Furthermore, patients with a prolonged stay had lower baseline SpO2 values (92.0 ± 3.6 vs. 93.5 ± 4.3; p < 0.001) and required higher rates of intervention in the emergency sector, including salbutamol (76.4%vs. 50.3%; p < 0.001) and oral corticosteroids (64.9%vs. 41.4%; p < 0.001). Conversely, patients with a stay ≤ 3 h showed higher rates of intravenous corticosteroid (43.8%vs. 30.1%; p = 0.005) and MgSO_4_ administration (41.4%vs. 25.5%; p < 0.001).

**Figure 1:**
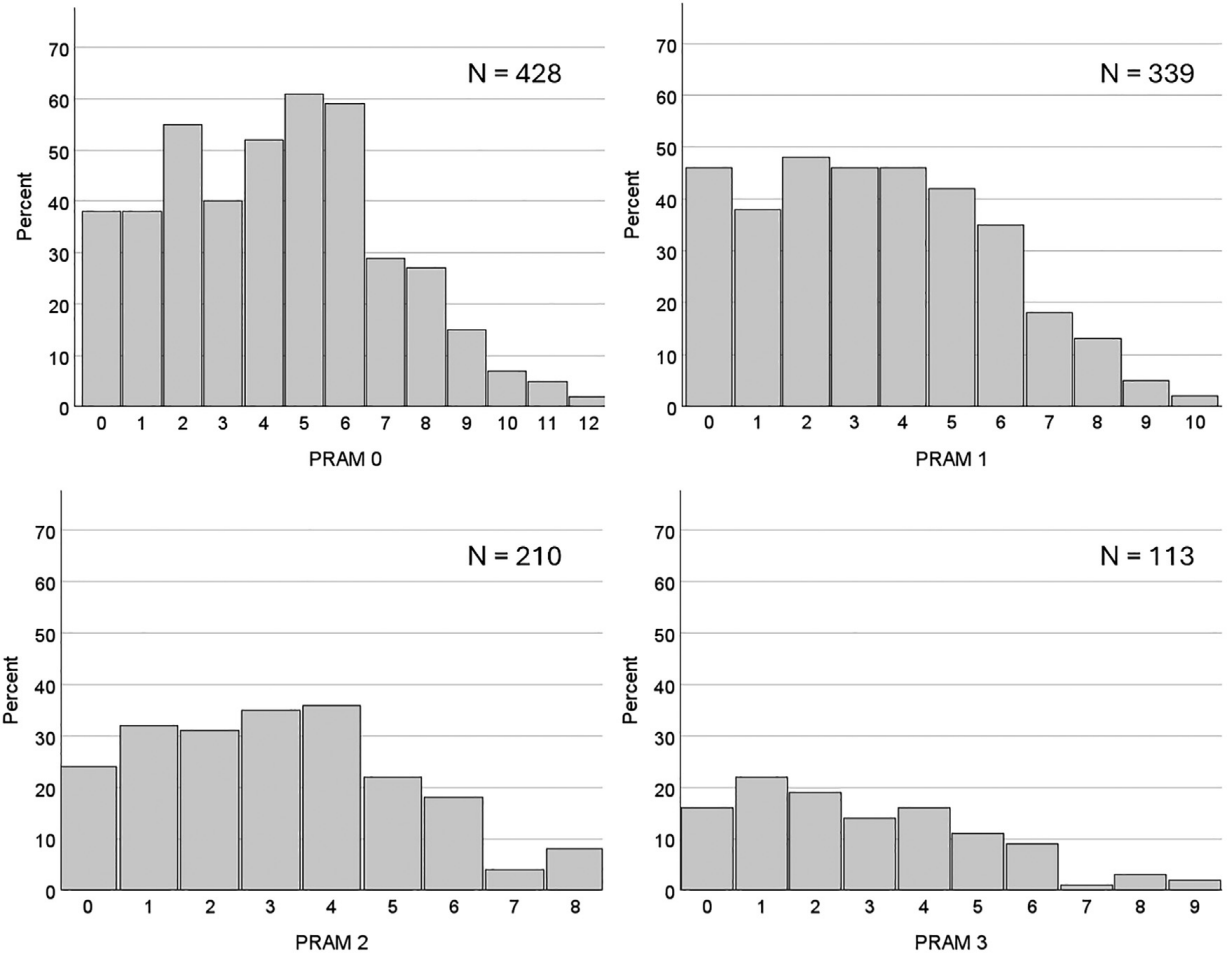
Histogram - distribution of PRAM scores.

At triage (PRAM 0), scores ranged from 0 to 12, concentrated predominantly in the intermediate values (4 to 6). According to the PRAM severity classification, 52.1% of the visits were mild, 41.1% moderate, and 6.8% severe. The distribution and progression of PRAM scores across all serial assessment points are illustrated in Figure 1.

Subsequent serial assessments documented a progressive decline in available data due to patients reaching a clinical endpoint. Mild cases predominated across all subsequent intervals: 66.1% at PRAM 1 (n = 339), 75.2% at PRAM 2 (n = 210), 77.0% at PRAM 3 (n = 113), and 72.7% at PRAM 4 (n = 11). Moderate cases represented 31.9%, 24.8%, 21.2%, and 18.2% of these cohorts, respectively, while severe cases accounted for 2.1% at PRAM 1, 0% at PRAM 2, 1.8% at PRAM 3, and 9.1% at PRAM 4. Missing data points sequentially reached 20.8%, 50.9%, 73.6%, and 97.4%, reflecting early discharge or hospital admission.

Regarding operational timelines, the median time to corticosteroid administration was 30 min (IQR: 15–100). The median time elapsed until a clinical decision was 4 h (IQR: 2–6) for patients discharged directly from the ED (n = 276) and 5 h (IQR: 3–8) for those who progressed to hospital admission (n = 150).

Regarding clinical outcomes, hospital admission was required for 150 patients (35.0%), while 278 (65.0%) were discharged directly from the emergency department. A prolonged stay in the service — defined as the interval exceeding three hours between admission and the final clinical decision recorded in the electronic medical record —occurred in 259 visits (60.5%). The distribution of these outcomes characterizes the clinical progression and severity of the studied visits within the emergency department.

### PRAM as a predictor of hospital admission

**Figure 2:**
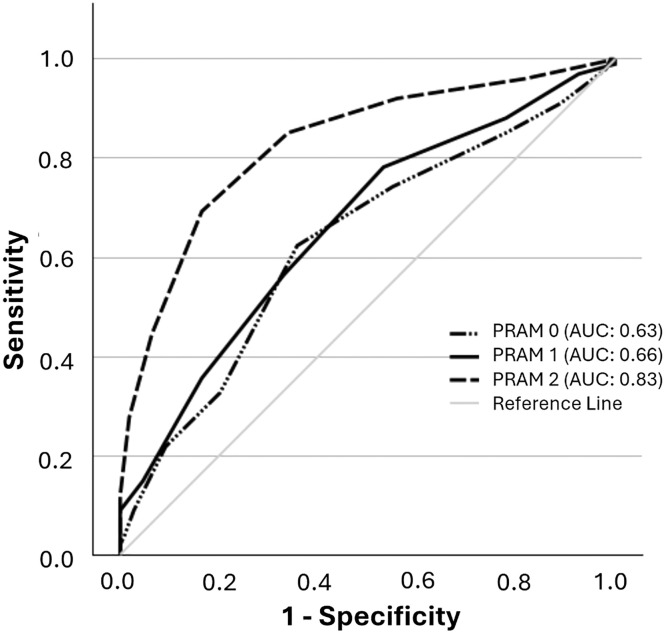
Combined ROC curves for predicting hospital admission at the first three hospital assessments: admission (PRAM 0), after the first hour (PRAM 1), and after the second hour (PRAM 2).

The discriminatory capacity of the PRAM score to predict hospital admission was evaluated using ROC curves across different time points. At baseline (PRAM 0), the score demonstrated good accuracy (AUC: 0.791; 95% CI: 0.745–0.836; p < 0.001), which remained consistent at the first hour (PRAM 1; AUC: 0.781; 95% CI: 0.731–0.832; p < 0.001). Predictive capacity optimized by the second hour of care (PRAM 2), yielding the highest discriminatory performance among the initial intervals (AUC: 0.829; 95% CI: 0.773–0.885; p < 0.001). These findings, illustrated in Figure 2, underscore that serial assessments post-initial therapeutic intervention offer progressively superior prognostic accuracy compared to a single static measurement at admission.

In later assessments, the score maintained high discriminatory capacity for hospital admission at both the third (PRAM 3; AUC: 0.918; 95% CI: 0.867–0.968; p < 0.001) and fourth hours (PRAM 4; AUC: 0.911; 95% CI: 0.725–1.000; p = 0.030). However, these high areas under the curve reflect an increasingly restricted, selective subset of patients under prolonged observation (n = 113 and n = 11, respectively). Due to this *'survival bias'* and wide confidence intervals, later metrics were interpreted strictly descriptively without additional graphical representations.

Regarding clinical thresholds, this study did not aim to establish a definitive normative cutoff point. PRAM coordinates were analyzed in an exploratory manner, demonstrating that values between 3 and 4 points provided a favorable balance between sensitivity and specificity, whereas higher scores expectedly maximized specificity at the expense of sensitivity. Thus, these thresholds should be utilized as exploratory clinical support to guide risk stratification rather than deterministic decision rules.

The association between clinical severity across sequential PRAM assessments and the time elapsed until the hospital admission decision was evaluated using Spearman's correlation. As presented in Table 2, no statistically significant correlation was observed at any time point, including admission (PRAM 0: spearman’s rho (*ρ*) = −0.105; p = 0.201), the first hour (PRAM 1: *ρ* = −0.020; p = 0.821), second hour (PRAM 2: *ρ* = 0.108; p = 0.284), third hour (PRAM 3: *ρ* = 0.116; p = 0.376), or fourth hour of care (PRAM 4: *ρ* = 0.224; p = 0.629). These results indicate that sequential PRAM scores were not linearly associated with the duration of stay required to solidify the final disposition for hospitalized patients (p > 0.05).

**Table 2:** Correlation between the PRAM score and the time to the hospital admission decision.

**Moment of PRAM assessment**	**Spearman's rho (*ρ*)**	**95% CI**	**p-value**	**N**
PRAM 0 (admission)	–0.105	–0.265 to 0.061	0.201	150
PRAM 1 (1st hour)	–0.020	–0.194 to 0.155	0.821	134
PRAM 2 (2nd hour)	0.108	–0.095 to 0.302	0.284	101
PRAM 3 (3rd hour)	0.116	–0.149 to 0.366	0.376	60
PRAM 4 (4th hour)	0.224	–0.653 to 0.845	0.629	7

Spearman's rho(ρ) = Spearman correlation coefficient. 95%CI: 95%confidence interval. p < 0.05 considered statistically significant.

### PRAM as a predictor of prolonged stay in the emergency department

The discriminatory capacity of the PRAM score to predict a prolonged length of stay was also evaluated using ROC curves, considering different moments of clinical assessment in the emergency department. The accuracy of the PRAM score in the second hour of care (PRAM 2) in predicting prolonged stay in the emergency department, defined as a time exceeding three hours until a clinical decision, showed moderate accuracy, with an area under the curve (AUC) of 0.677 (95% CI: 0.602–0.751; p < 0.001), indicating statistically significant performance in discriminating between patients with and without prolonged stay in the service (Supplementary Fig. 1). Although this analysis was applied to the other PRAM scores, they did not reach statistical significance.

### Association between PRAM and prolonged stay in the emergency department

The association between clinical severity assessed by PRAM classification and prolonged emergency department stay is shown in Table 3. At admission, visits classified as moderate/severe had a higher frequency of stay > 3 h compared with those classified as mild (71.2%vs. 50.7%; p < 0.001). After the first hour of care, this association remained statistically significant, with a higher proportion of prolonged stay among visits with moderate/severe PRAM compared with mild PRAM (80.9%vs. 69.6%; p = 0.033). At the second hour, although prolonged stay was frequent in both groups, no statistically significant association was observed between PRAM classification and prolonged stay (94.2%vs. 89.9%; p = 0.430). At the third and fourth hours, prolonged stay was observed in nearly all visits, and ORs were not estimable because of sparse/zero cells.

**Table 3:** Association between PRAM classification at different moments of care and prolonged emergency department stay (> 3 h).

**Moment of PRAM**	**Classification**	**Total**	**Stay**		p*	Non-adjusted OR (95% CI)*	**p**⁎⁎	**Adjusted OR (95% CI)**⁎⁎
			**≤ 3h**	**> 3h**				
Admission	*Mild*	223	110 (49.3%)	113 (50.7%)				
	*Moderate/Severe*	205	59 (28.8%)	146 (71.2%)	<0.001	2.45 (1.62 – 3.71)	0.012	1.95 (1.16 – 3.28)
1st hour	*Mild*	224	68 (30.4%)	156 (69.6%)				
	*Moderate/Severe*	115	22 (19.1%)	93 (80.9%)	0.033	1.85 (1.05 – 3.25)	0.044	1.94 (1.02 – 3.69)
2nd hour	*Mild*	158	16 (10.1%)	142 (89.9%)				
	*Moderate/Severe*	52	3 (5.8%)	49 (94.2%)	0.430	1.63 (0.48 – 5.49)	0.398	1.73 (0.49 – 6.13)
3rd hour	*Mild*	87	1 (1.1%)	86 (98.9%)				
	*Moderate/Severe*	26	0 (0.0%)	26 (100%)	-	-	-	-
4th hour	*Mild*	8	0 (0.0%)	8 (100%)				
	*Moderate/Severe*	3	0 (0.0%)	3 (100%)	-	-	-	-

Data are expressed as n (%). Percentages were calculated within rows, using the total number of visits in each PRAM classification category as the denominator.

PRAM was classified as mild (≤ 4 points) or moderate/severe (≥ 5 points). Prolonged stay was defined as a stay > 3 h in the emergency department. Odds ratios (ORs) compare moderate/severe PRAM with mild PRAM as the reference category.

⁎ P-values, ORs, and 95% confidence intervals (95% CIs) were obtained from unadjusted generalized linear mixed models (GLMMs).

⁎⁎ P-values, ORs, and 95% CIs were obtained from multivariable GLMMs adjusted for previous ICU admission for asthma, SpO₂, and magnesium use. All models included a random intercept for patient to account for repeated visits. For the 3rd- and 4th-hour PRAM assessments, ORs were not estimable because of sparse/zero cells.

In multivariable models adjusted for previous ICU admission for asthma, SpO₂, and magnesium use, moderate/severe PRAM at admission remained independently associated with prolonged emergency department stay (adjusted OR 1.95, 95% CI 1.16–3.28; p = 0.012). A similar finding was observed for PRAM at the first hour (adjusted OR 1.94, 95% CI 1.02–3.69; p = 0.044). In contrast, PRAM at the second hour was not significantly associated with prolonged stay after adjustment (adjusted OR 1.73, 95% CI 0.49–6.13; p = 0.398).

## Discussion

The findings of this study suggest the applicability of PRAM as a reliable predictive tool for risk stratification in the pediatric emergency setting, consistent with previous investigations [6,11]. The score demonstrated satisfactory discriminatory capacity for hospital admission at triage, with optimized accuracy during sequential reassessments (particularly at the second hour). This pattern corroborates the value of dynamic therapeutic monitoring described by Gouin et al. [10] and Alnaji et al. [11] Additionally, the association between higher initial scores and prolonged emergency stays underscores the direct impact of baseline clinical severity on pediatric patient flow, aligning with research on the standardization of acute asthma management [4,7].

The present data support the role of PRAM as an objective tool for risk stratification, reducing the subjectivity associated with isolated physical examinations [8,9]. The baseline accuracy observed (AUC: 0.791) aligns with literature demonstrating a direct relationship between higher scores and hospital admission [6,11], supporting GINA guidelines regarding objective severity assessments to guide initial management [2]. Crucially, PRAM should function as a quantitative substrate to support diagnostic reasoning rather than a deterministic algorithm replacing individualized medical judgment.

Furthermore, serial analysis proved essential. The superior predictive accuracy at the second hour (PRAM 2; AUC: 0.829) over admission indicates that clinical response post-initial treatment offers a more reliable prognosis than static triage measurements. The subsequent reduction in evaluable patients at PRAM 3 and 4 reflects the natural dynamics of the department — where responders are progressively discharged, leaving a highly selective, therapeutically resistant cohort. This pattern represents a clinical *'survival bias'* rather than selection bias, validating the score’s sensitivity in tracking bronchospasm resolution under real-world conditions [10].

Regarding prolonged stay in the emergency department, defined as a stay exceeding three hours, PRAM demonstrated moderate but statistically significant predictive capacity (AUC 0.677 for PRAM 2; 95% CI: 0.602–0.751; p < 0.001). Although there was a statistically significant association between moderate/severe scores and longer length of stay in the initial assessments, the lower accuracy for this outcome, when compared to the prediction of admission, denotes the multifactorial nature of length of stay. It is important to recognize that both the length of stay and the decision for hospital admission are influenced by factors beyond isolated respiratory severity. Organizational factors, such as bed availability and administrative flow, as well as social variables —including the family's distance from the hospital and parental comfort with home management — play a significant role in the final clinical disposition, a phenomenon also observed in other pediatric respiratory conditions [13] and standardized admission criteria.

A notable and counterintuitive finding was the absence of a statistically significant correlation between sequential PRAM scores and the time elapsed until the admission decision (p > 0.05). This indicates that clinical severity alone does not accelerate disposition timelines. Instead, it suggests that defining therapeutic failure requires protocol-driven observation periods regardless of initial presentation, a process further influenced by institutional operational inertia. Consequently, while PRAM accurately identifies patients requiring admission, it does not modify the time needed to solidify this decision, which remains dependent on sustained therapeutic response.

Clinically, these results support the integration of PRAM into emergency flow management. The high specificity of elevated scores at the second hour can justify early hospital bed requests for this high-risk subgroup, optimizing department throughput. Furthermore, systematic score implementation establishes a standardized language among the multidisciplinary team, enhancing patient safety during care transitions in alignment with national and international guidelines [1,2,5].

This study presents significant strengths, including a robust sample size (n = 428) and a longitudinal evaluation of the score in a real-world setting. Analyzing ROC curves at multiple sequential time points addresses a critical gap in the literature often restricted to cross-sectional baseline measurements [6,9,11]. Additionally, distinguishing between clinical disposition (admission) and operational throughput (length of stay) provides a comprehensive view of how asthma exacerbations impact healthcare delivery.

Conversely, certain limitations apply. The single-center design may reflect localized flow characteristics, limiting direct generalizability. Furthermore, the progressive reduction of available data at later stages — exceeding 70% missing records at PRAM 3 and 4 — restricted inferential statistics for these intervals. This decay represents an intrinsic clinical *'survival bias'*, where the remaining cohort represents a highly selective subgroup with slower clinical resolution or greater severity. Consequently, metrics for PRAM 3 and PRAM 4 (n = 11) must be interpreted strictly as exploratory. Finally, the absence of structural confounders, such as emergency department occupancy rates or socioeconomic determinants during care, prevents a more refined analysis of non-clinical factors influencing prolonged stays.

Future developments in this line of research demand the execution of multicenter studies designed to validate flow protocols guided by specific PRAM cutoff points, mainly emphasizing the measurement of the concrete impact on reducing operational costs and hospital length of stay. Simultaneously, investigating the score's interoperability with emerging biomarkers constitutes strategic frontiers for the evolution of pediatric asthma management, paving the way for the transition from a purely clinical assessment to a precision medicine model in the pediatric urgency/emergency setting.

In conclusion, the findings of this study provide additional evidence supporting PRAM as a dynamic risk stratification tool in pediatric emergency asthma exacerbations. Serial assessment proved to be superior to isolated measurement at admission, reflecting therapeutic response and the need for admission more accurately. Although its ability to predict operational outcomes is limited by organizational factors, the score demonstrates consistent utility in clinical practice and can contribute to the standardization of assessment and care decision-making.

## Ethics committee approval

CAAE: 69784423.0.0000.0097.

## Declaration of generative AI and AI-assisted technologies in the manuscript preparation process

During the preparation of this work, the authors used Google Gemini in order to translate the manuscript into English and adjust the formatting to meet the journal's guidelines. After using this tool/service, the authors reviewed and edited the content as needed and take full responsibility for the content of the published article.

## Funding

This research did not receive any specific grant from funding agencies in the public, commercial, or not-for-profit sectors.

## Conflicts of interest

The authors declare no conflicts of interest.

## Data Availability

The data that support the findings of this study are available from the corresponding author.

## Appendix Supplementary materials

Image, application 1
