# Supplementary material for: The pediatric respiratory assessment measure as a predictor of hospitalization in the management of asthma exacerbations: an observational study
Source: J Pediatr (Rio J). 2026 Jul 24;102(5):101583. doi: 10.1016/j.jped.2026.101583 (PMC13444265; doi:10.1016/j.jped.2026.101583)
Supplement: Supplementary file 1 [file mmc1.docx]

**JPED-D-26-00170_ Supplementary Material**

**Supplementary Figure 1** ROC Curve for predicting prolonged stay (> 3 h) with PRAM 2.

**Sensitivity analysis**

To assess the impact of losses across reassessment time points, a sensitivity analysis was performed to compare visits with and without PRAM reassessments at subsequent time points. Visits with available PRAM 1, PRAM 2, and PRAM 3 assessments differed from those without reassessment, particularly regarding initial severity and clinical outcomes. They had lower SpO₂, higher frequency of moderate/severe PRAM at admission, greater use of acute asthma therapies, and higher frequencies of hospital admission and prolonged emergency department stay. These findings indicate that missing PRAM reassessments were not random but were related to the clinical course and duration of emergency department observation. Therefore, analyses involving PRAM reassessments should be interpreted as conditional on reassessment availability, whereas PRAM at admission represents the most robust assessment because it was available for all visits (Supplementary Table 1).

**Supplementary Table 1** Comparison of clinical characteristics and outcomes according to availability of PRAM reassessments (sensitivity analysis).

| **Variables** | **Evaluation of PRAM 1** | | | **Evaluation of PRAM 2** | | | **Evaluation of PRAM 3** | | |
| --- | --- | --- | --- | --- | --- | --- | --- | --- | --- |
|  | **No (N=89)** | **Yes (N=339)** | **p*** | **No (N=218)** | **Yes (N=210)** | **p*** | **No** | **Yes** | **p*** |
| Age (years) (mean ± sd) | 6.8 ± 3.6 | 6.2 ± 3.0 | 0.124 | 6.5 ± 3.4 | 3.4 ± 6.0 | 0.267 | 6.4 ± 3.3 | 3.3 ± 6.0 | 0.273 |
| Male sex, n (%) | 41 (46.1%) | 196 (57.8%) | 0.059 | 118 (54.1%) | 119 (56.7%) | 0.523 | 180 (57.1%) | 57 (50.4%) | 0.230 |
| Duration of previous symptoms (days) (median; IQR) | 2 (1 – 4) | 1 (1 – 3) | 0.007 | 1 (1 – 3) | 1 (1 – 3) | 0.114 | 1 (1 – 3) | 2 (1 – 3) | 0.368 |
| Associated upper respiratory tract infection, n (%) | 47 (52.8%) | 161 (47.5%) | 0.370 | 109 (50%) | 99 (47.1%) | 0.558 | 156 (49.5%) | 52 (46%) | 0.522 |
| Personal history of atopy, n (%) | 46 (51.7%) | 188 (55.5%) | 0.505 | 114 (52.3%) | 120 (57.1%) | 0.326 | 162 (51.4%) | 72 (63.7%) | 0.028 |
| Family history of atopy, n (%) | 30 (33.7%) | 132 (39.1%) | 0.385 | 85 (39%) | 77 (36.8%) | 0.653 | 128 (40.6%) | 34 (30.4%) | 0.060 |
| Previous use of salbutamol, n (%) | 52 (58.4%) | 206 (60.8%) | 0.661 | 126 (57.8%) | 132 (62.9%) | 0.295 | 182 (57.8%) | 76 (67.3%) | 0.085 |
| Previous use of inhaled corticosteroids  (> 2 weeks), n (%) | 39 (43.8%) | 138 (40.7%) | 0.613 | 95 (43.6%) | 82 (39%) | 0.342 | 135 (42.9%) | 42 (37.2%) | 0.294 |
| Use of inhaled corticosteroids in current crisis, n (%) | 31 (34.8%) | 100 (29.5%) | 0.317 | 82 (37.6%) | 49 (23.3%) | 0.002 | 117 (37.1%) | 14 (12.4%) | <0.001 |
| Previous use of montelukast, n (%) | 29 (32.6%) | 103 (30.4%) | 0.663 | 82 (37.6%) | 50 (23.8%) | 0.003 | 114 (36.2%) | 18 (15.9%) | <0.001 |
| Previous use of antibiotics, n (%) | 35 (39.3%) | 110 (32.4%) | 0.220 | 89 (40.8%) | 56 (26.7%) | 0.003 | 127 (40.3%) | 18 (15.9%) | <0.001 |
| Previous hospital admission for asthma, n (%) | 37 (41.6%) | 137 (40.5%) | 0.856 | 96 (44%) | 78 (37.3%) | 0.160 | 131 (41.6%) | 43 (38.4%) | 0.556 |
| Previous ICU admission for asthma, n (%) | 31 (34.8%) | 94 (27.8%) | 0.202 | 82 (37.6%) | 43 (20.6%) | <0.001 | 112 (35.6%) | 13 (11.6%) | <0.001 |
| SPO2 | 95.4 ± 3.8 | 91.9 ± 3.7 | <0.001 | 93.9 ± 3.9 | 91.2 ± 3.6 | <0.001 | 93.1 ± 4.0 | 91.2 ± 3.4 | <0.001 |
| SALBUTAMOL | 36 (40.4%) | 247 (72.9%) | <0.001 | 117 (53.7%) | 166 (79%) | <0.001 | 182 (57.8%) | 101 (89.4%) | <0.001 |
| IPRATRÓPRIO | 28 (31.5%) | 123 (36.3%) | 0.416 | 76 (34.9%) | 75 (35.7%) | 0.846 | 112 (35.6%) | 39 (34.5%) | 0.843 |
| CTC ORAL | 32 (36%) | 206 (60.8%) | <0.001 | 94 (43.1%) | 144 (68.6%) | <0.001 | 154 (48.9%) | 84 (74.3%) | <0.001 |
| CTC EV | 34 (38.2%) | 118 (34.8%) | 0.534 | 88 (40.4%) | 64 (30.5%) | 0.037 | 126 (40%) | 26 (23%) | 0.002 |
| MgSO4 | 30 (33.7%) | 106 (31.3%) | 0.644 | 81 (37.2%) | 55 (26.2%) | 0.018 | 118 (37.5%) | 18 (15.9%) | <0.001 |
| PRAM 0 moderate/severe | 12 (13.5%) | 193 (56.9%) | <0.001 | 70 (32.1%) | 135 (64.3%) | <0.001 | 131 (41.6%) | 74 (65.5%) | <0.001 |
| Admissão hospitalar | 16 (18%) | 134 (39.5%) | <0.001 | 49 (22.5%) | 101 (48.1%) | <0.001 | 90 (28.6%) | 60 (53.1%) | <0.001 |
| Estadia prolongada (>3h) | 10 (11.2%) | 249 (73.5%) | <0.001 | 68 (31.2%) | 191 (91%) | <0.001 | 147 (46.7%) | 112 (99.1%) | <0.001 |

*ICU: intensive care unit; sd: standard deviation; IQR: interquartile interval*

*Data expressed as mean ± standard deviation, median (interquartile interval), or frequency and percentages calculated based on the total number of valid cases for each variable;
*Generalized Linear Mixed Model (GLMM).*
